# Genetic risk of obesity as a modifier of associations between neighbourhood environment and body mass index: an observational study of 335 046 UK Biobank participants

**DOI:** 10.1136/bmjnph-2020-000107

**Published:** 2020-10-05

**Authors:** Kate E Mason, Luigi Palla, Neil Pearce, Jody Phelan, Steven Cummins

**Affiliations:** 1 Department of Public Health, Policy and Systems, University of Liverpool, Liverpool, UK; 2 Department of Non-communicable Disease Epidemiology, London School of Hygiene and Tropical Medicine, London, UK; 3 Department of Medical Statistics, London School of Hygiene and Tropical Medicine, London, UK; 4 Department of Global Health, University of Nagasaki, Nagasaki, Japan; 5 Department of Infection Biology, London School of Hygiene and Tropical Medicine, London, UK; 6 Department of Public Health, Environments and Society, London School of Hygiene and Tropical Medicine, London, UK

**Keywords:** dietary patterns, malnutrition

## Abstract

**Background:**

There is growing recognition that recent global increases in obesity are the product of a complex interplay between genetic and environmental factors. However, in gene-environment studies of obesity, ‘environment’ usually refers to individual behavioural factors that influence energy balance, whereas more upstream environmental factors are overlooked. We examined gene-environment interactions between genetic risk of obesity and two neighbourhood characteristics likely to be associated with obesity (proximity to takeaway/fast-food outlets and availability of physical activity facilities).

**Methods:**

We used data from 335 046 adults aged 40–70 in the UK Biobank cohort to conduct a population-based cross-sectional study of interactions between neighbourhood characteristics and genetic risk of obesity, in relation to body mass index (BMI). Proximity to a fast-food outlet was defined as distance from home address to nearest takeaway/fast-food outlet, and availability of physical activity facilities as the number of formal physical activity facilities within 1 km of home address. Genetic risk of obesity was operationalised by weighted Genetic Risk Scores of 91 or 69 single nucleotide polymorphisms (SNP), and by six individual SNPs considered separately. Multivariable, mixed-effects models with product terms for the gene-environment interactions were estimated.

**Results:**

After accounting for likely confounding, the association between proximity to takeaway/fast-food outlets and BMI was stronger among those at increased genetic risk of obesity, with evidence of an interaction with polygenic risk scores (p=0.018 and p=0.028 for 69-SNP and 91-SNP scores, respectively) and in particular with a SNP linked to *MC4R* (p=0.009), a gene known to regulate food intake. We found very little evidence of gene-environment interaction for the availability of physical activity facilities.

**Conclusions:**

Individuals at an increased genetic risk of obesity may be more sensitive to exposure to the local fast-food environment. Ensuring that neighbourhood residential environments are designed to promote a healthy weight may be particularly important for those with greater genetic susceptibility to obesity.

**What this paper adds**

- We examined gene-environment interactions between genetic risk of obesity and two neighbourhood characteristics likely to be associated with obesity.
- Our study suggests that individuals at increased genetic risk of obesity may be more sensitive to living near a fast-food outlet, but we found little evidence that genetic risk interacts with neighbourhood availability of physical activity facilities to influence BMI.
- Ensuring that neighbourhood residential environments are designed to promote a healthy weight may be particularly important for those with greater genetic susceptibility to obesity.

## Background

Obesity has a heritable component,^1^ but the rapid rise in global obesity prevalence suggests an important role for environmental influences.^2^ However, individuals may have differing physiological or behavioural responses to the increasingly ‘obesogenic’ environment, suggesting that a complex interplay between genetic and non-genetic factors affects weight.^3 4^

Advances in genotyping technologies have enabled the investigation of gene-environment (G×E) interactions.^4^ For obesity outcomes, the ‘environment’ in G×E studies is often operationalised as the lifestyle or behavioural factors that influence energy balance,^5^ rather than more upstream features of the built and social environments—the settings where behavioural ‘choices’ are made and constrained. Some recent studies have examined interactions between genetic risk and birth cohort as a means of capturing exposure to an increasingly obesogenic environment in very broad terms,^6–8^ but there has been limited investigation of specific features of the environment that might plausibly interact with genetic risk,^9–11^ despite a number of ‘socioecological’ environmental factors long being recognised in social epidemiology as potentially important determinants of weight status.

The residential neighbourhood environment comprises many features that potentially influence energy balance. These include the proximity, density and relative proportions of healthy and unhealthy food retailers,^12–14^ and resources for physical activity (PA), such as leisure centres, swimming pools, gyms and sports fields.^15–17^ Other neighbourhood features linked to energy balance include walkability, access to public transport and local resources such as public parks and greenspace.^18 19^ If genetic risk of obesity modifies the influence of these neighbourhood exposures, then we would expect to observe differential effects of the residential environment on body mass index (BMI) according to the level of genetic risk. There are various models of G×E interaction that may be in operation. Under the dominant diathesis–stress model, the influence of an obesogenic environment is expected to be strongest in people with high genetic risk due to increased sensitivity to external factors. Conversely, the beneficial effects of a health-promoting environment may be strongest among people with low genetic risk, who can maximise their genetic ‘advantage’ within a healthier environment, whereas those at greater risk express a higher BMI phenotype regardless of environmental factors. Under Belsky’s alternative differential susceptibility model, individuals with obesity-linked genetic variants would have higher BMI than their counterparts in an obesogenic environment but a lower BMI than their counterparts in a more health-promoting environment.^5 20^

In this study, we use the UK Biobank cohort to examine whether genetic risk of obesity modifies the effect of two residential environment exposures likely to influence BMI: proximity to fast-food and availability of formal PA facilities. We operationalise genetic risk in two ways. First, using polygenic risk scores derived from single-nucleotide polymorphisms (SNPs) linked to BMI, and second, using the individual SNPs most strongly linked to BMI and thought to be involved in diet or PA pathways.

## Methods

### Data

We used baseline data from UK Biobank.^21^ Data were potentially available from 502 656 individuals who visited 22 UK Biobank assessment centres across the UK between 2006 and 2010. Individuals aged 40–69 years living within 25 miles of an assessment centre and listed on National Health Service patient registers were invited to participate.

Linked to UK Biobank is the UK Biobank Urban Morphometric Platform (UKBUMP), a high-resolution spatial database of objectively measured characteristics of the physical environment surrounding each participant’s residential address, derived from multiple national spatial datasets.^22^ Environmental measures include densities of various land uses and proximity to various health-relevant resources. Measures for this study are available for 96% of the UK Biobank sample.

Genome-wide genetic data are available for 488 363 participants. Genetic data are missing from 3% of the sample as insufficient DNA was extracted from blood samples for genotyping assays. SNP genotypes not directly assayed were imputed. Procedures used to derive the genetic data and undertake quality assurance are reported in Bycroft *et al*.^23^ Genetic data for the relevant SNPs were downloaded, decrypted and linked to participant IDs to facilitate analysis.

### Outcome

BMI (kg/m^2^) was calculated from weight and height measurements collected by trained staff using standard procedures.^21^ The variable was normally distributed and analysed as a continuous outcome variable.

### Neighbourhood exposures

We examined interactions between genetic risk and two neighbourhood characteristics likely to influence BMI: availability of formal PA facilities (number of indoor and outdoor sporting and leisure facilities within a 1 km street network distance of an individual’s home) and fast-food proximity (distance in metres to nearest takeaway/fast-food outlet). Greater neighbourhood availability of PA facilities may influence BMI through increased opportunities for PA, and greater distances from home to fast-food outlets may influence BMI by reducing access to fast food.^24 25^ We chose these measures after finding in prior, published analyses that both were associated with BMI in the expected direction—that is, living further from a fast-food outlet, or having more PA facilities near home, was associated with having a lower BMI.^15^ Both exposures are measured at the level of the individual and were analysed as continuous variables, with higher values of each (more facilities; greater distance to nearest fast-food outlet) representing lower exposure. Due to the ranges and positively skewed distribution of these variables, number of PA facilities was capped at 15 (<1% recoded from >15) and distance to the nearest fast-food outlet was log-transformed (base 10) such that regression coefficients were interpreted as the mean difference in BMI associated with a 10-fold increase in distance to the nearest fast-food outlet, for example, 100 m to 1 km.

### Genetic Risk Scores and individual SNPs

A recent genome-wide association study (GWAS) identified 97 SNPs associated with BMI.^26^ We constructed a Genetic Risk Score (GRS) based on 91 of these SNPS, excluding 6 SNPs identified elsewhere^27^ as being in linkage disequilibrium with other included SNPs (rs17001654, rs2075650 and rs9925964) or having pleiotropic effects (rs11030104, rs3888190 and rs13107325), both of which may produce bias in associations between the GRS and the outcome, and in interaction analyses.^28^ We also constructed an alternative GRS, the same as the one used by Tyrrell and colleagues^27^ in a study of UK Biobank participants of White British ancestry, in which they tested interactions between genetic risk and behavioural exposures using a GRS derived from 69 of the SNPs identified in the recent GWAS. Their GRS excluded SNPs from secondary meta-analyses of studies of regional, sex-stratified or non-European-descent populations,^26^ and one SNP (rs2033529) that was unavailable at the time of their study. Full lists of the SNPs included in each of the 91-SNP and 69-SNP risk scores are provided in the online supplemental table 1. The GRSs were constructed by summing the number of BMI-increasing alleles across the set of 69 or 91 loci and weighting the allele count at each SNP by its published effect size.^26^ For imputed SNP genotypes we used the imputed allelic dosages.

10.1136/bmjnph-2020-000107.supp1 Supplementary data

From the literature, we identified individual SNPs with a well-established link to obesity, likely via dietary intake and with the largest published effect sizes (rs1558902, rs6567160 and rs13021737, markers of the *FTO*, *MC4R* and *TMEM18* genes, respectively),^1 26^ and three SNPs recently linked to PA (rs13078960, rs10938397 and rs7141420, markers of *CADM2*, *GNPDA2 and NRXN3,* respectively).^29 30^ To support the primary GRS analyses, we tested for interactions between the number of BMI-increasing alleles at each of these loci and each neighbourhood variable. We hypothesise that if any G×E interactions are observed for these SNPs, then those SNPs implicated in dietary behaviour will only interact with the fast-food environment, and those implicated in PA behaviour will only interact with the PA environment.

### Covariates

Models were adjusted for potential confounding by age, sex, educational attainment, household income, employment status, area deprivation (Townsend score), urban/non-urban status, and neighbourhood residential density and mutually adjusted for the other neighbourhood exposure as the two are correlated. We also corrected for population stratification by adjusting for the first 10 of 40 UK Biobank-provided genetic ancestry principal components from a genome-wide principal component analysis (PCA) of UK Biobank’s genetic data.^23^

### Statistical analysis and analytic sample

Accounting for the nested structure of the data (individuals within assessment areas) and after comparing model fit against simpler model specifications, we used mixed-effects models with a random intercept and a random coefficient for the neighbourhood exposure and assuming an unstructured variance/covariance matrix. Models included an interaction term between the primary exposure (neighbourhood environment) and the potential effect modifier (GRS), with both analysed as continuous variables. The p value for the additive interaction term was interpreted as the strength of evidence of effect modification by genetic risk, and to summarise the model results, we used the margins command in Stata to estimate BMI difference per unit change in the environmental exposure, for each quintile of genetic risk. These marginal predicted values of BMI associated with different levels of each neighbourhood exposure were plotted for the top and bottom quintile of genetic risk, to visualise observed effect heterogeneity according to genetic risk. We then repeated the analysis for each of the six individual SNPs for which we hypothesised that interactions would exist with one but not the other environmental exposure. Analyses used complete case data only, and were restricted to UK Biobank participants of white British ancestry (defined by concordant self-report and PCA results for white British/Caucasian ancestry) for the primary analyses because the smaller GRS was limited to SNPs associated with BMI in analyses of individuals with European ancestry. The sample size for the primary analysis was 335 046. All analyses were performed using Stata SE V.14.2.

### Sensitivity analyses

As the 91-SNP GRS included SNPs associated with BMI in populations of non-European descent, we undertook a sensitivity analysis that tested for an interaction with the 91-SNP GRS in a sample unrestricted by ethnicity to test generalisability to the wider source population. To explore the possibility that results might be biased by latent genetic structure in the sample—a concern regarding genetic analyses involving UK Biobank^31^—we also performed sensitivity analyses in which models were adjusted for all 40 genetic ancestry principal components and for birth location. Finally, although weighting of the polygenic risk scores is appropriate due to the varying degree to which each SNP is associated with BMI, we performed sensitivity analyses using an unweighted version of each GRS. Evidence of a G×E interaction using unweighted scores is expected to be weaker due to dilution of the effects of the more influential SNPs.

### Ethics

UK Biobank has ethical approval from the North West Multicentre Research Ethics Committee (reference 16/NW/0274), the Patient Information Advisory Group, and the Community Health Index Advisory Group. Additional ethical approval for the specific study was obtained from the London School of Hygiene and Tropical Medicine’s Research Ethics Committee in September 2016 (reference 11897).

## Results

The sample was 52.2% female individuals, with a mean age of 56.5 years (range 40–70 years at baseline). The mean BMI was 27.4 kg/m^2^ (SD=4.7), median distance to the nearest fast-food outlet was 1171 m and median number of PA facilities within 1 km of the home was 1. The sample characteristics are summarised in table 1.

**Table 1:** Characteristics of primary sample and top and bottom quintile of 91-SNP Genetic Risk Score

	91-SNP Genetic Risk Score		Total sample
	Quintile 1 (lowest risk of obesity)	Quintile 5 (highest risk of obesity)	
Total number of participants	64 269	69 577	335 046
BMI (kg/m^2^), mean (SD)	26.5 (4.3)	28.3 (5.1)	27.4 (4.7)
Distance to nearest fast-food outlet (m), median (IQR)	1172 (634–2302)	1169 (626–2290)	1171 (630–2301)
Number of PA facilities within 1 km of home address, median (IQR)	1 (0–3)	1 (0–3)	1 (0–3)
Age (y), mean (SD)	56.5 (8.0)	56.5 (8.0)	56.5 (8.0)
Sex (female), n (%)	33 876 (52.7)	35 923 (51.6)	17 4872 (52.2)
Income (£), n (%)			
Less than 18 000	14 154 (22.0)	15 734 (22.6)	74 556 (22.3)
18 000–30 999	16 497 (25.7)	18 270 (26.3)	86 917 (25.9)
31 000–51 999	17 013 (26.5)	18 374 (26.4)	88 721 (26.5)
52 000–100 000	13 269 (20.7)	13 738 (19.8)	67 908 (20.3)
Greater than 100 000	3336 (5.2)	3461 (5.0)	16 944 (5.1)
Education, n (%)			
College or university degree	21 462 (33.4)	22 412 (32.2)	110 153 (32.9)
A levels/AS levels or equivalent	7635 (11.9)	7961 (11.4)	39 017 (11.7)
O levels/GCSEs or equivalent	14 262 (22.2)	15 779 (22.7)	74 966 (22.4)
CSEs or equivalent	3500 (5.5)	3933 (5.7)	18 722 (5.6)
NVQ or HND or HNC or equivalent	4266 (6.6)	4782 (6.9)	22 892 (6.8)
Other professional qualifications	3302 (5.1)	3527 (5.1)	16 954 (5.1)
None of the above	9842 (15.3)	11 183 (16.1)	52 342 (15.6)
Employment status, n (%)			
Paid employment or self-employed	38 217 (59.5)	41 326 (59.4)	199 280 (59.5)
Retired	21 330 (33.2)	23 096 (33.2)	111 113 (33.2)
Unable to work	1756 (2.7)	2055 (3.0)	9457 (2.8)
Unemployed	769 (1.2)	878 (1.3)	4238 (1.3)
Home duties/carer/student/other	2197 (3.4)	2222 (3.2)	10 958 (3.3)
Urbanicity (% urban dwelling)	54 560 (84.9)	59 018 (84.8)	284 471 (84.9)
Area deprivation,* mean (SD)	−1.6 (2.9)	−1.6 (2.9)	−1.6 (2.9)
Residential density,† median (IQR)	1794 (1041–2934)	1801 (1043–2911)	1798 (1044–2918)

*2001 Townsend index score.

†Residential address points per 1 km street network buffer around home address.

AS, Advanced Study; BMI, body mass index; CSE, Certificate of Secondary Education; GCSE, General Certificate of Secondary Education; HNC, Higher National Certificate; HND, Higher National Diploma; NVQ, National Vocational Qualification; PA, physical activity; SNP, single-nucleotide polymorphism.

**Figure 1:**
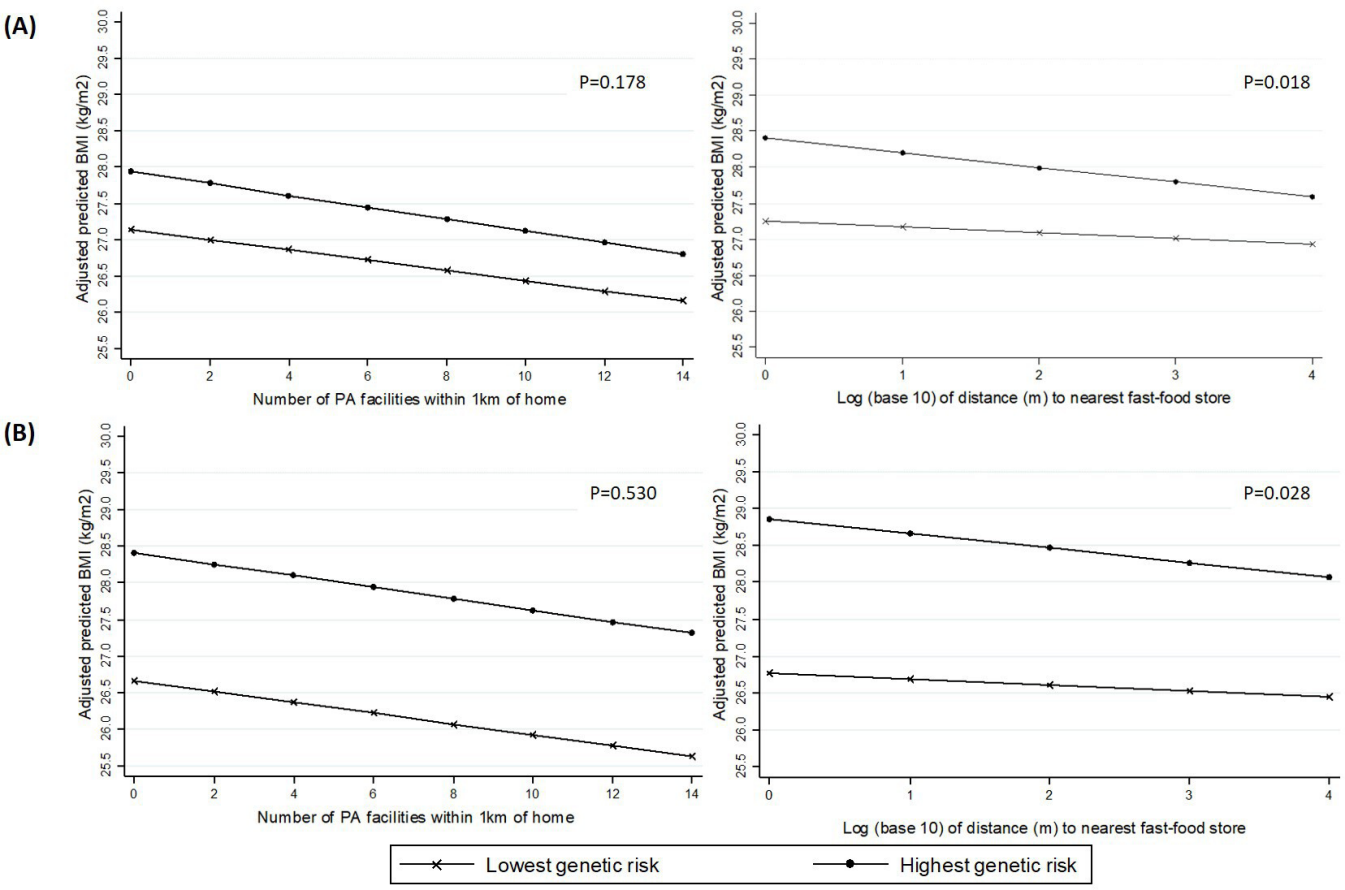
Associations between neighbourhood variables and BMI in the highest and lowest quintiles of genetic risk, based on (A) 69-SNP Genetic Risk Score, and (B) 91-SNP Genetic Risk Score. BMI, body mass index; PA, physical activity; SNP, single-nucleotide polymorphism,

Using the two alternative weighted GRSs, we observed evidence of an interaction between fast-food proximity and genetic risk (p=0.028 for the 91-SNP GRS, p=0.018 for the 69-SNP GRS). The magnitude of the estimated effect between fast-food proximity and BMI was small at all levels of genetic risk, but increased monotonically as genetic risk increased, suggesting a dose response. In the highest quintile of genetic risk of obesity (based on the 91-SNP GRS), each 10-fold increase in distance to the nearest fast-food store was associated with a 0.194 kg/m^2^ lower mean BMI (95% CI −0.326 to 0.062), which was twice the magnitude of association in the lowest risk quintile (β=−0.081; 95% CI −0.213 to 0.052) (table 2; figure 1).

**Table 2:** Associations between neighbourhood variables and BMI, by quintile of genetic risk based on 91-SNP and 69-SNP GRSs

	91-SNP GRS			69-SNP GRS		
	Quintile of genetic risk	Mean BMI difference for unit increase in neighbourhood exposure	P for interaction	Quintile of genetic risk	Mean BMI difference for unit increase in neighbourhood exposure	P for interaction
Fast-food proximity*† (unit change = 10-fold increase in distance (m) to nearest fast-food outlet)	Q1	−0.081 (−0.213 to 0.052)	0.028	Q1	−0.080 (−0.214 to 0.055)	0.018
	Q2	−0.115 (−0.239 to 0.009)		Q2	−0.117 (−0.243 to 0.009)	
	Q3	−0.137 (−0.259 to −0.014)		Q3	−0.140 (−0.264 to −0.017)	
	Q4	−0.158 (−0.282 to −0.035)		Q4	−0.164 (−0.289 to −0.039)	
	Q5	−0.194 (−0.326 to −0.062)		Q5	−0.204 (−0.337 to −0.070)	
Availability of PA facilities*‡ (unit change = one additional facility)	Q1	−0.074 (−0.100 to −0.047)	0.530	Q1	−0.070 (−0.097 to −0.044)	0.178
	Q2	−0.075 (−0.101 to −0.049)		Q2	−0.074 (−0.099 to −0.048)	
	Q3	−0.076 (−0.101 to −0.050)		Q3	−0.076 (−0.101 to −0.050)	
	Q4	−0.077 (−0.103 to −0.051)		Q4	−0.078 (−0.103 to −0.052)	
	Q5	−0.078 (−0.105 to −0.052)		Q5	−0.081 (−0.106 to −0.054)	

*Regression models were adjusted for age (years), sex (male/female), highest education level attained (degree; A level or equivalent; O level/GCSE or equivalent; CSE or equivalent; NVQ/HND/HNC; other professional qualification; none of the above), annual household income (<£18 000; £18,000–£30 999; £31000–£51 999; £52 000–£100 000; >£100 000), employment status (paid work, retired, unable to work, unemployed, other), area deprivation (Townsend score), urbanicity (urban/non-urban), neighbourhood residential density (count of residential features within a 1 km street network buffer of home address, log transformed).

†Also adjusted for availability of PA facilities.

‡Also adjusted for fast-food proximity.

BMI, body mass index; CSE, Certificate of Secondary Education; GRS, genetic risk score; HNC, Higher National Certificate; HND, Higher National Diploma; NVQ, National Vocational Qualification; PA, physical activity; SNP, single-nucleotide polymorphism.

There was less evidence that the association between the availability of PA facilities and BMI was modified by genetic risk. The magnitude of the association between the number of formal PA facilities within 1 km of home and BMI was similar at all levels of genetic risk, and although effect estimates did increase slightly with increasing genetic risk, differences between risk groups were small and we were unable to find evidence of interaction with either the 91-SNP GRS (p=0.530) or 69-SNP GRS (p=0.178). For both environmental exposures, the results obtained from the two different weighted GRSs were substantively similar, but evidence of an interaction with the 69-SNP GRS was somewhat stronger (table 2; figure 1). The plots in figure 1 also demonstrate that the BMI difference between the highest and lowest risk quintiles is greater for the 91-SNP GRS than the 69-SNP GRS, reflecting the fact that the larger GRS captures more of the genetic variation in BMI.

**Figure 2:**
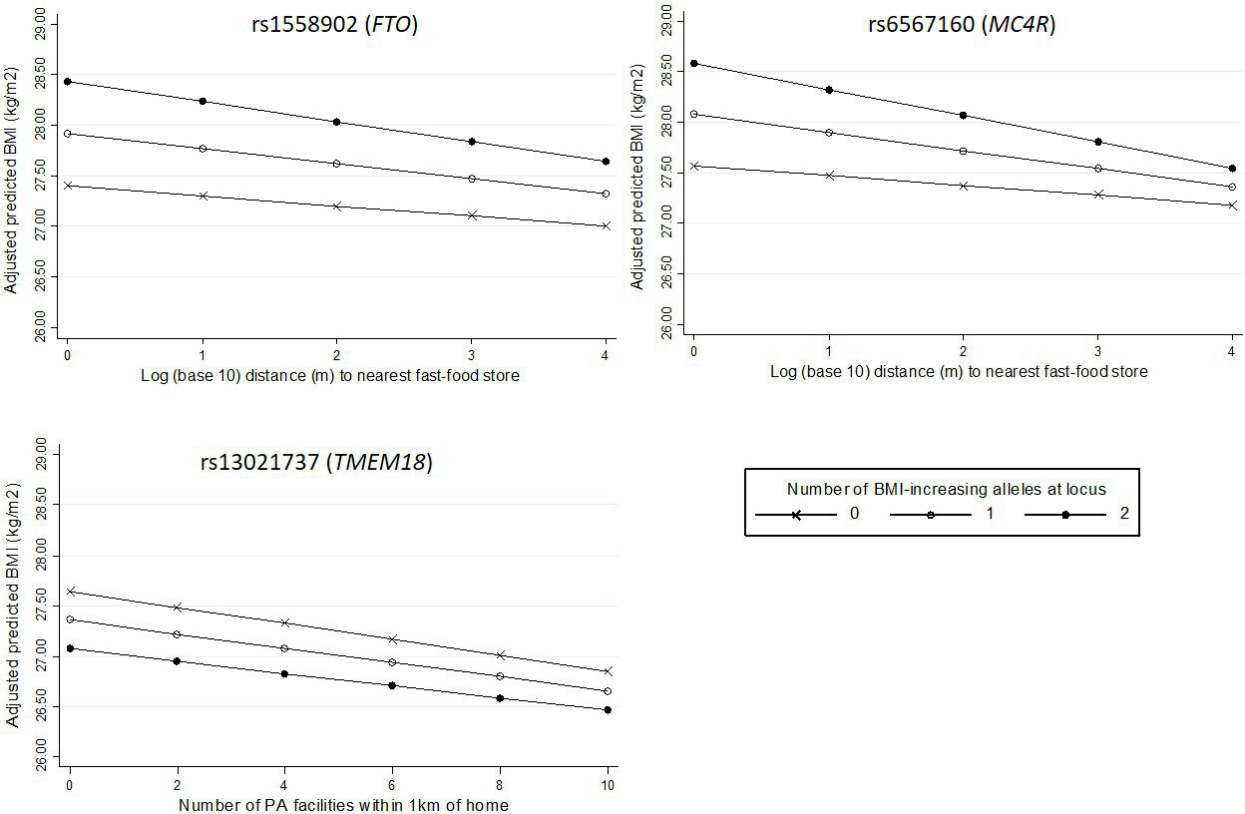
Associations between neighbourhood variables and BMI according to number of risk alleles at individual SNPs where p_interaction_<0.10 (rs1558902 and rs6567160 for fast-food proximity; rs13021737 for availability of PA facilities). BMI, body mass index; PA, physical activity; SNP, single-nucleotide polymorphism.

Examination of interactions between neighbourhood variables and specific SNPs revealed strong evidence of one interaction: with the marker of *MC4R*, which encodes the melanocortin-4 receptor previously shown to be important in the regulation of food intake. Among people homozygous for the high-risk allele at the marker of *MC4R*, each 10-fold increase in distance to the nearest fast-food store was associated with a 0.258 kg/m^2^ lower BMI, compared with only a 0.096 kg/m2 difference per 10-fold increase in distance among people with no risk alleles at this locus ((p_interaction_=0.009, table 3; figure 2). There was some evidence of an interaction between fast-food proximity and rs1558902, the marker of the *FTO* gene (p=0.067), where again the higher risk group showed a stronger association between fast-food proximity and BMI. We also observed weak evidence of a G×E interaction between the availability of PA facilities and rs13021737 (in the *TMEM18* gene) (p=0.076). In this case, increased genetic risk slightly attenuated the association between the availability of PA facilities and BMI (figure 2).

**Table 3:** Associations between neighbourhood variables and BMI, testing interaction with number of risk alleles at selected loci

	P_interaction_	Homozygous low risk (0 risk alleles)	Heterozygous (one risk allele)	Homozygous high risk (two risk alleles)
	rs1558902 (*FTO*)			
Fast-food proximity	0.067	−0.099 (−0.198 to −0.001)	−0.148 (−0.238 to −0.059)	−0.197 (−0.305 to −0.088)
Availability of PA facilities	0.933	−0.077 (−0.104 to −0.050)	−0.077 (−0.103 to −0.051)	−0.076 (−0.104 to −0.049)
	rs6567160 (*MC4R*)			
Fast-food proximity	0.009	−0.096 (−0.188 to −0.003)	−0.177 (−0.271 to −0.083)	−0.258 (−0.386 to −0.130)
Availability of PA facilities	0.606	−0.078 (−0.104 to −0.051)	−0.075 (−0.102 to −0.049)	−0.073 (−0.103 to −0.043)
	rs13021737 (*TMEM18*)			
Fast-food proximity	0.993	−0.135 (−0.226 to −0.043)	−0.135 (−0.234 to −0.036)	−0.135 (−0.279 to 0.008)
Availability of PA facilities	0.076	−0.080 (−0.106 to −0.053)	−0.071 (−0.098 to −0.043)	−0.061 (−0.093 to −0.030)
	rs13078960 (*CADM2*)			
Fast-food proximity	0.114	−0.159 (−0.252 to −0.066)	−0.108 (−0.205 to −0.010)	−0.056 (−0.192 to 0.081)
Availability of PA facilities	0.419	−0.076 (−0.102 to −0.049)	−0.079 (−0.106 to −0.053)	−0.083 (−0.114 to −0.053)
	rs10938397 (*GNPDA2*)			
Fast-food proximity	0.328	−0.115 (−0.215 to −0.015)	−0.141 (−0.230 to −0.052)	−0.167 (−0.274 to −0.061)
Availability of PA facilities	0.694	−0.076 (−0.102 to −0.049)	−0.077 (−0.103 to −0.051)	−0.079 (−0.106 to −0.051)
	rs7141420 *(NRXN3)*			
Fast-food proximity	0.520	−0.152 (−0.257 to −0.048)	−0.135 (−0.227 to −0.043)	−0.118 (−0.224 to −0.012)
Availability of PA facilities	0.125	−0.071 (−0.097 to −0.044)	−0.077 (−0.102 to −0.051)	−0.083 (−0.110 to −0.056)

PA, physical activity; SNP, single-nucleotide polymorphism.

In sensitivity analyses, interactions between fast-food proximity and genetic risk were—as expected—weaker when the GRSs were not weighted by the effect sizes of the component SNPs, with mean differences in BMI more similar across levels of genetic risk than we observed using the weighted score (online supplemental table 2). Expanding the sample to include non-white ethnicities, we observed slightly increased p values for the interaction terms but otherwise no substantive difference from the primary analysis (online supplemental table 3). For all models, the impact of adjusting for 40 rather than 10 genetic ancestry principal components was negligible, whereas some attenuation of the interaction between fast-food proximity and polygenic risk occurred when adjusting for birth location (online supplemental table 4).

## Discussion

In UK Biobank, we found evidence that genetic risk of obesity modified sensitivity to the neighbourhood food environment, though effects were small and further studies are needed to replicate and extend this research. We found that living closer to a fast-food outlet was more strongly associated with higher BMI among people at higher genetic risk of obesity, whereas for those at the lowest genetic risk of obesity, distance to the nearest fast-food outlet did not appear to be associated with BMI. Evidence of a dose response was apparent. In contrast, an overall negative association between neighbourhood availability of PA facilities and BMI varied very little across levels of polygenic risk.

Given the large sample size and only moderately small p values for the G×E interactions, it is possible the observed G×E interaction for fast-food proximity using polygenic risk scores may not be substantively meaningful. Lending further weight to these GRS results, however, was the stronger evidence of an interaction between fast-food proximity and a specific SNP near *MC4R*, a gene known to be involved in the regulation of food intake.^32^ Previous research has linked *MC4R* specifically to binge eating^33^ although this remains contested.^34^ We also observed some evidence of a possible interaction with a SNP marker of *FTO*, a gene with well-established links to obesity. Although *FTO* has long been recognised as an obesity-associated locus and has been implicated in central nervous system regulation of appetite, its exact function remains poorly understood.^1^ In a study of gene–diet interactions, GRSs for BMI were found to be associated with fried food consumption, and, consistent with our results, individual loci in or near both *MC4R* and *FTO* contributed to this.^35^ The limited evidence we found of an interaction between genetic risk and the PA environment is consistent with findings from a recent study in adolescents that found that the availability of recreation facilities did not contribute to the attenuation by PA of genetic risk of obesity.^29^ Although overall genetic risk of obesity did not interact with the PA environment in our study, the weaker association we observed between the availability of PA facilities and BMI in those with more risk alleles at the *TMEM18* locus suggests that some specific SNPs might. Further examination of other SNPs is warranted. Lack of interaction with specific SNPs might be explained by the pathways they influence being less sensitive to environmental exposures. As the functional pathways by which most BMI-associated loci influence BMI remain poorly understood, it is difficult to speculate further.

Stronger evidence for interactions with specific SNPs highlights the lack of specificity of polygenic risk scores. Although useful in exploratory studies, grouping all SNPs statistically associated with a complex phenotype such as BMI into a single score, regardless of the function of the genes they represent, may dilute or obscure important interactions. Scores based on known or putative biological mechanisms may prove more valuable, particularly for elucidating causal relationships. We observed very similar results for both the 69-SNP and 91-SNP GRSs, although the smaller GRS yielded stronger evidence of an interaction. It may be that the additional SNPs in the larger GRS diluted the interaction due to being associated with BMI only in some population subgroups and some having been linked to BMI only in more ethnically diverse populations than our primary sample.^26^

We have reported elsewhere that the main association between fast-food proximity and BMI in UK Biobank may be attenuated due to measurement error in the exposure^15^ and because the exposure does not account for other, healthier elements of the food environment.^36^ Compared with other measures of the fast-food environment, proximity measures may also produce more conservative estimates of association with relevant outcomes.^37^ In a regional subsample of UK Biobank, others have recently improved on this measurement of the food environment and found stronger associations.^38^ In this study, where the main effect sizes are relatively small, even the reasonably strong interaction effects we observed translate to small differences between high-risk and low-risk groups. However, given the likely measurement error and the distal and complex nature of the relationships under investigation, detecting even weak associations and small differences might point to potentially important processes. Other studies have reported evidence of a G×E interaction between the genetic risk of obesity and birth cohort,^6–8^ with this interpreted as evidence that recent increases in the ‘obesogenicity’ of our environments increase the susceptibility of those with a genetic predisposition to become obese. Here we examined two characteristics of neighbourhood environments likely to be obesogenic but others may also interact with genetic risk. For example, G×E interactions have recently been reported for neighbourhood walkability and obesity,^10^ and neighbourhood deprivation and BMI.^9^ Given that unhealthy characteristics of neighbourhoods often cluster together,^39^ the combined effects of multiple ‘obesogenic’ features on those at increased genetic risk of obesity may be substantial. Further research with data on the actual use of local environmental resources may enable us to better understand these observed interactions. Our findings with regard to a gene–food environment interaction were consistent with a diathesis–stress model rather than a differential susceptibility model, in that although individuals with a higher GRS were somewhat more sensitive to the fast-food environment, they were not more advantaged (ie, with lower BMI) than their lower GRS counterparts when not living near a fast-food store. However, this distinction may be better tested using a more comprehensive measure of the food environment.

Our novel study provides preliminary evidence for a potentially important G×E interaction, but confirmatory studies are required. Another recent study found a strong G×E interaction between the genetic risk of obesity and socioeconomic status, and although our analyses are adjusted for several socioeconomic indicators, if there remains any residual confounding by socioeconomic status, then it may be contributing to the G×E interactions we observed. Geographical genetic structure in the sample remains a risk, even after adjustment for ancestry components and geography. Such a structure may induce spurious associations with polygenic risk scores in particular.^31^ In sensitivity analyses, we found that adjustment for additional ancestry principal components had a negligible impact on the strength of evidence for the G×E interactions we tested, but evidence for genetic interaction with fast-food proximity was slightly weaker following adjustment for birth location. Further investigation of the effect of the residual genetic structure in the sample is warranted. G×E interactions are also sensitive to the scaling of environmental variables, and the power to detect a G×E interaction can depend on the main effect sizes, and distribution and measurement quality of the genetic and environmental variables.^40^ Studies using UK Biobank are also at risk of selection bias due to a low response rate and a healthier, wealthier sample than the general UK population, and this may have influenced our results.^41^ The large sample size also increases the risk of generating statistically ‘significant’ findings that are of no substantive importance. It is therefore important that these analyses are replicated in other samples at lower risk of these biases and that future studies investigate potential sources of bias in greater depth.

It is widely accepted that environmental factors are important in explaining the recent rise in the global prevalence of overweight and obesity. In this study, we find evidence suggesting that people at higher genetic risk of obesity may be more sensitive to the residential fast-food environment. If confirmed by other studies, these findings suggest that ensuring neighbourhood residential environments are designed to promote a healthy weight may be particularly important for those with genetic susceptibility to obesity.
