# Supplementary material for: Genetic risk of obesity as a modifier of associations between neighbourhood environment and body mass index: an observational study of 335 046 UK Biobank participants
Source: BMJ Nutr Prev Health. 2020 Oct 5;3(2):247–55. doi: 10.1136/bmjnph-2020-000107 (PMC7841812; doi:10.1136/bmjnph-2020-000107)
Supplement: Supplementary data [file bmjnph-2020-000107supp001.pdf]

SUPPLEMENTARY MATERIAL

Genetic risk of obesity as a modifier of associations between neighbourhood environment and body mass index: an observational study of 335 046 UK Biobank participants

Kate E Mason, Luigi Palla, Neil Pearce, Jody Phelan, Steven Cummins

Supplementary Table 1. SNPs included in each polygenic risk score

SNP	Chr	Position	Gene	BMI-increasing allele	Effect size (β coefficient per effect allele, SD units of BMI)	Included in 69-SNP risk score	Included in 91-SNP risk score	Reason for exclusion[1,2]
**rs1558902**	16	52,361,075	*FTO*	A	0.0818	Yes	Yes	
**rs17024393**	1	109,956,211	*GNAT2*	C	0.0658	Yes	Yes	
**rs13021737**	2	622,348	*TMEM18*	G	0.0601	Yes	Yes	
**rs6567160**	18	55,980,115	*MC4R*	C	0.0556	Yes	Yes	
**rs11847697**	14	29,584,863	*PRKD1*	T	0.0492	Yes	Yes	
**rs16851483**	3	142,758,126	*RASA2*	T	0.0483	Yes	Yes	
**rs543874**	1	176,156,103	*SEC16B*	G	0.0482	Yes	Yes	
**rs13107325**	4	103,407,732	*SLC39A8*	T	0.0477	No	No	Possible pleiotropy
**rs1516725**	3	187,306,698	*ETV5*	C	0.0451	Yes	Yes	
**rs2207139**	6	50,953,449	*TFAP2B*	G	0.0447	Yes	Yes	
**rs11030104**	11	27,641,093	*BDNF*	A	0.0414	No	No	Possible pleiotropy
**rs12446632**	16	19,842,890	*GPRC5B*	G	0.0403	Yes	Yes	
**rs10938397**	4	44,877,284	*GNPDA2*	G	0.0402	Yes	Yes	
**rs7899106**	10	87,400,884	*GRID1*	G	0.0395	Yes	Yes	
**rs2287019**	19	50,894,012	*QPCTL*	C	0.0360	Yes	Yes	
**rs11727676**	4	145,878,514	*HHIP*	T	0.0358	Yes	Yes	
**rs16907751**	8	81,538,012	*ZBTB10*	C	0.0350	No	Yes	Identified in secondary meta-analyses only
**rs12429545**	13	53,000,207	*OLFM4*	A	0.0334	Yes	Yes	
**rs3101336**	1	72,523,773	*NEGR1*	C	0.0334	Yes	Yes	
**rs2245368**	7	76,446,079	*DTX2P1*	C	0.0317	Yes	Yes	
**rs7138803**	12	48,533,735	*BCDIN3D*	A	0.0315	Yes	Yes	
**rs16951275**	15	65,864,222	*MAP2K5*	T	0.0311	Yes	Yes	
**rs3888190**	16	28,796,987	*ATP2A1*	A	0.0309	No	No	Possible pleiotropy
**rs11191560**	10	104,859,028	*NT5C2*	C	0.0308	Yes	Yes	
**rs10182181**	2	25,003,800	*ADCY3*	G	0.0307	Yes	Yes	
**rs11057405**	12	121,347,850	*CLIP1*	G	0.0307	Yes	Yes	

Mason KE, *et al. bmjnph* 2021; 3:e000107. doi: 10.1136/bmjnph-2020-000107

SNP	Chr	Position	Gene	BMI-increasing allele	Effect size (β coefficient per effect allele, SD units of BMI)	Included in 69-SNP risk score	Included in 91-SNP risk score	Reason for exclusion[1,2]
**rs17001654**	4	77,348,592	*SCARB2*	G	0.0306	No	No	Linkage disequilibrium
**rs9581854**	13	26,915,782	*MTIF3*	T	0.0298	Yes	Yes	
**rs13078960**	3	85,890,280	*CADM2*	G	0.0297	Yes	Yes	
**rs3810291**	19	52,260,843	*ZC3H4*	A	0.0283	Yes	Yes	
**rs13191362**	6	162,953,340	*PARK2*	A	0.0277	Yes	Yes	
**rs3817334**	11	47,607,569	*MTCH2*	T	0.0262	Yes	Yes	
**rs2112347**	5	75,050,998	*POC5*	T	0.0261	Yes	Yes	
**rs2075650**	19	50,087,459	*TOMM40*	A	0.0258	No	No	Linkage disequilibrium
**rs10968576**	9	28,404,339	*LINGO2*	G	0.0249	Yes	Yes	
**rs17094222**	10	102,385,430	*HIF1AN*	C	0.0249	Yes	Yes	
**rs2121279**	2	142,759,755	*LRP1B*	T	0.0245	Yes	Yes	
**rs12566985**	1	74,774,781	*FPGT*	G	0.0242	Yes	Yes	
**rs7141420**	14	78,969,207	*NRXN3*	T	0.0235	Yes	Yes	
**rs7903146**	10	114,748,339	*TCF7L2*	C	0.0234	No	Yes	Not included in Tyrrell et al's GRS (reason unclear)
**rs13201877**	6	137,717,234	*IFNGR1*	G	0.0233	No	Yes	Identified in secondary meta-analyses only
**rs10132280**	14	24,998,019	*STXBP6*	C	0.0230	Yes	Yes	
**rs1016287**	2	59,159,129	*LINC01122*	T	0.0229	Yes	Yes	
**rs657452**	1	49,362,434	*AGBL4*	A	0.0227	Yes	Yes	
**rs758747**	16	3,567,359	*NLRC3*	T	0.0225	Yes	Yes	
**rs17405819**	8	76,969,139	*HNF4G*	T	0.0224	Yes	Yes	
**rs205262**	6	34,671,142	*C6orf106*	G	0.0221	Yes	Yes	
**rs7599312**	2	213,121,476	*ERBB4*	G	0.0220	Yes	Yes	
**rs11165643**	1	96,696,685	*PTBP2*	T	0.0218	Yes	Yes	
**rs12286929**	11	114,527,614	*CADM1*	G	0.0217	Yes	Yes	
**rs7243357**	18	55,034,299	*GRP*	T	0.0217	Yes	Yes	
**rs12401738**	1	78,219,349	*FUBP1*	A	0.0211	Yes	Yes	
**rs17203016**	2	207,963,763	*CREB1*	G	0.0210	No	Yes	Identified in secondary meta-analyses only

Mason KE, *et al. bmjnph* 2021; 3:e000107. doi: 10.1136/bmjnph-2020-000107

SNP	Chr	Position	Gene	BMI-increasing allele	Effect size (β coefficient per effect allele, SD units of BMI)	Included in 69-SNP risk score	Included in 91-SNP risk score	Reason for exclusion[1,2]
**rs4256980**	11	8,630,515	*TRIM66*	G	0.0209	Yes	Yes	
**rs11126666**	2	26,782,315	*KCNK3*	A	0.0207	Yes	Yes	
**rs12885454**	14	28,806,589	*PRKD1*	C	0.0207	Yes	Yes	
**rs2650492**	16	28,240,912	*SBK1*	A	0.0207	Yes	Yes	
**rs1167827**	7	75,001,105	*HIP1*	G	0.0202	Yes	Yes	
**rs9914578**	17	1,951,886	*SMG6*	G	0.0201	No	Yes	Identified in secondary meta-analyses only
**rs2365389**	3	61,211,502	*FHIT*	C	0.0200	Yes	Yes	
**rs2176598**	11	43,820,854	*HSD17B12*	T	0.0198	Yes	Yes	
**rs1460676**	2	164,275,935	*FIGN*	C	0.0197	No	Yes	Identified in secondary meta-analyses only
**rs2820292**	1	200,050,910	*NAV1*	C	0.0195	Yes	Yes	
**rs17724992**	19	18,315,825	*PGPEP1*	A	0.0194	Yes	Yes	
**rs1000940**	17	5,223,976	*RABEP1*	G	0.0192	Yes	Yes	
**rs2033732**	8	85,242,264	*RALYL*	C	0.0192	Yes	Yes	
**rs9925964**	16	31,037,396	*KAT8*	A	0.0192	No	No	Linkage disequilibrium
**rs9641123**	7	93,035,668	*CALCR*	C	0.0191	No	Yes	Identified in secondary meta-analyses only
**rs2033529**	6	40,456,631	*TDRG1*	G	0.0190	No	Yes	Not included in Tyrrell et al's GRS because unavailable
**rs1928295**	9	119,418,304	*TLR4*	T	0.0188	Yes	Yes	
**rs3849570**	3	81,874,802	*GBE1*	A	0.0188	Yes	Yes	
**rs6091540**	20	50,521,269	*ZFP64*	C	0.0188	No	Yes	Identified in secondary meta-analyses only
**rs9400239**	6	109,084,356	*FOXO3*	C	0.0188	Yes	Yes	
**rs9374842**	6	120,227,364	*LOC285762*	T	0.0187	No	Yes	Identified in secondary meta-analyses only
**rs6804842**	3	25,081,441	*RARB*	G	0.0185	Yes	Yes	
**rs12940622**	17	76,230,166	*RPTOR*	G	0.0182	Yes	Yes	
**rs29941**	19	39,001,372	*KCTD15*	G	0.0182	Yes	Yes	
**rs7164727**	15	70,881,044	*LOC100287559*	T	0.0180	No	Yes	Identified in secondary meta-analyses only
**rs4740619**	9	15,624,326	*C9orf93*	T	0.0179	Yes	Yes	
**rs1528435**	2	181,259,207	*UBE2E3*	T	0.0178	Yes	Yes	

SNP	Chr	Position	Gene	BMI-increasing allele	Effect size (β coefficient per effect allele, SD units of BMI)	Included in 69-SNP risk score	Included in 91-SNP risk score	Reason for exclusion[1,2]
**rs11583200**	1	50,332,407	*ELAVL4*	C	0.0177	Yes	Yes	
**rs3736485**	15	49,535,902	*DMXL2*	A	0.0176	Yes	Yes	
**rs1441264**	13	78,478,920	*MIR548A2*	A	0.0175	No	Yes	Identified in secondary meta-analyses only
**rs10733682**	9	128,500,735	*LMX1B*	A	0.0174	Yes	Yes	
**rs6477694**	9	110,972,163	*EPB41L4B*	C	0.0174	Yes	Yes	
**rs11688816**	2	62,906,552	*EHBP1*	G	0.0172	Yes	Yes	
**rs9540493**	13	65,103,705	*MIR548X2*	A	0.0172	No	Yes	Identified in secondary meta-analyses only
**rs2080454**	16	47,620,091	*CBLN1*	C	0.0168	No	Yes	Identified in secondary meta-analyses only
**rs1808579**	18	19,358,886	*C18orf8*	C	0.0167	Yes	Yes	
**rs977747**	1	47,457,264	*TAL1*	T	0.0167	No	Yes	Identified in secondary meta-analyses only
**rs6465468**	7	95,007,450	*ASB4*	T	0.0166	No	Yes	Identified in secondary meta-analyses only
**rs2836754**	21	39,213,610	*ETS2*	C	0.0164	No	Yes	Identified in secondary meta-analyses only
**rs7239883**	18	38,401,669	*LOC284260*	G	0.0164	No	Yes	Identified in secondary meta-analyses only
**rs7715256**	5	153,518,086	*GALNT10*	G	0.0163	No	Yes	Identified in secondary meta-analyses only
**rs4787491**	16	29,922,838	*INO80E*	G	0.0159	No	Yes	Identified in secondary meta-analyses only
**rs492400**	2	219,057,996	*USP37*	C	0.0158	No	Yes	Identified in secondary meta-analyses only
**rs2176040**	2	226,801,046	*LOC646736*	A	0.0141	No	Yes	Identified in secondary meta-analyses only

Note: This table is derived from Locke et al (2015), with additional information from Tyrrell et al (2017).

Supplementary Table 2. Results of sensitivity analyses using unweighted genetic risk scores

	69-SNP unweighted imputed GRS (n=332,127)			***91-SNP unweighted imputed GRS (n=326,604)***		
	Quintile of genetic risk	Mean BMI difference for unit increase in neighbourhood exposure	P-interaction	Quintile of genetic risk	Mean BMI difference for unit increase in neighbourhood exposure	P-interaction
**Fast-food proximity** (log (base 10) of distance (m) to nearest fast-food outlet)	Q1	-0.094 (-0.229, 0.042)		Q1	-0.103 (-0.237, 0.030)	
	Q2	-0.123 (-0.250, 0.004)		Q2	-0.130 (-0.254, -0.005)	
	Q3	-0.143 (-0.268, -0.017)	0.064	Q3	-0.146 (-0.269, -0.023)	0.116
	Q4	-0.162 (-0.289, -0.034)		Q4	-0.161 (-0.286, -0.037)	
	Q5	-0.191 (-0.327, -0.055)		Q5	-0.186 (-0.319, -0.053)	
**Availability of PA facilities** (number of formal PA facilities within 1km of home address)	Q1	-0.069 (-0.096, -0.042)		Q1	-0.069 (-0.096, -0.042)	
	Q2	-0.073 (-0.099, -0.047)		Q2	-0.073 (-0.099, -0.047)	
	Q3	-0.075 (-0.101, -0.050)	0.119	Q3	-0.076 (-0.102, -0.050)	0.110
	Q4	-0.078 (-0.104, -0.052)		Q4	-0.078 (-0.104, -0.052)	
	Q5	-0.081 (-0.108, -0.054)		Q5	-0.082 (-0.109, -0.055)	

Mason KE, *et al. bmjnph* 2021; 3:e000107. doi: 10.1136/bmjnph-2020-000107

Supplementary Table 3. Results of sensitivity analyses using an expanded sample including observations from UK Biobank participants of non-White ethnicities

		91-SNP weighted imputed GRS (N=374,339)	
	Quintile of genetic risk	Mean BMI difference for unit increase in neighbourhood exposure	P-interaction
**Fast-food proximity** (log (base 10) of distance (m) to nearest fast-food outlet)	Q1	-0.086 (-0.208, 0.036)	0.026
	Q2	-0.119 (-0.233, -0.005)	
	Q3	-0.140 (-0.252, -0.028)	
	Q4	-0.161 (-0.274, -0.047)	
	Q5	-0.195 (-0.318, -0.073)	
**Availability of PA facilities** (number of formal PA facilities within 1km of home address)	Q1	-0.068 (-0.095, -0.041)	0.197
	Q2	-0.071 (-0.097, -0.046)	
	Q3	-0.072 (-0.098, -0.046)	
	Q4	-0.074 (-0.100, -0.048)	
	Q5	-0.077 (-0.104, -0.050)	

Supplementary Table 4. Results of sensitivity analyses additionally adjusting for extra 30 genetic ancestry principal components and birth location

	**Fast-food proximity**		**Availability of PA facilities**	
**P-for-interaction**	Adjusted for 40 ancestral PCs	Adjusted for 40 PCs & place of birth	Adjusted for 40 ancestral PCs	Adjusted for 40 PCs & place of birth
69-SNP GRS	0.015	0.030	0.385	0.395
91-SNP GRS	0.028	0.069	0.536	0.550
rs1558902 (FTO)	0.066	0.088	0.928	0.904
rs6567160 (MC4R)	0.009	0.025	0.617	0.766
rs13021737 (TMEM18)	0.999	0.967	0.081	0.063
rs13078960 (CADM2)	0.108	0.142	0.407	0.361
rs10938397 (GNPDA2)	0.306	0.301	0.732	0.964
rs7141420 (NRXN3)	0.519	0.665	0.114	0.155

REFERENCES

1. Tyrrell J, Wood AR, Ames RM, et al. Gene–obesogenic environment interactions in the UK Biobank study. *Int J Epidemiol*. 2017:dyw337. doi:10.1093/ije/dyw337.

2. Locke AE, Kahali B, Berndt SI, et al. Genetic studies of body mass index yield new insights for obesity biology. *Nature*. 2015;518(7538):197-206. doi:10.1038/nature14177.
